# Intra- and inter-rater reliability of medial elbow joint space width on static ultrasound images: a cross-sectional reliability study

**DOI:** 10.1186/s13102-025-01469-9

**Published:** 2025-12-13

**Authors:** Shotaro Teruya, Shizuka Akahori, Ryuhei Michinobu, Hiromitsu Tsuge, Kazuhiro Ikeda, Yasukazu Totoki, Akira Ikumi, Yuichi Yoshii

**Affiliations:** 1 Department of Orthopaedic Surgery, Institute of Medicine, University of Tsukuba, 1-1-1 Tennodai, Tsukuba, Ibaraki 305-8575 Japan; 2 Graduate School of Science and Technology, University of Tsukuba, 1-1-1 Tennodai, Tsukuba, Ibaraki 305-8571 Japan; 3 Department of Orthopaedic Surgery, Tokyo Medical University Ibaraki Medical Center, 3- 20-1 Chuo, Ami, Ibaraki 300-0395 Japan

**Keywords:** Ultrasound examination, Elbow joint, Measurement reliability, Operator experience, Intra-rater reliability, Inter-rater agreement

## Abstract

**Background:**

Quantifying the reproducibility of ultrasound measurements of the medial elbow joint space is essential for interpreting changes over time and for comparing raters. However, measurement reliability under controlled, acquisition-independent conditions remains limited. Herein, we aimed to quantify the intra-rater repeatability and inter-rater agreement for static medial elbow ultrasound measurements exclusively focusing on the analytical (measurement) component in healthy elbows.

**Methods:**

In this cross-sectional reliability study, 130 high-quality static ultrasound images from healthy elbows (13 elbows × 10 images each) were acquired by a single experienced examiner using standardized limb positioning and evaluated twice by seven orthopedic surgeons with varying ultrasound experience. The medial elbow joint space width was defined as the narrowest distance between the distal medial edge of the humeral trochlea and the proximal edge of the ulnar trochlear notch. Measurements were blinded and repeated following a ≥ 2-week interval. Reliability was assessed using the following intraclass correlation coefficients: ICC(1,1) for intra-rater repeatability and ICC(2,1) for inter-rater agreement, with error metrics expressed as the standard error of measurement (SEM) and the minimal detectable change at 95% confidence (MDC95). In addition, the mean, standard deviation, and range were calculated to provide reference values.

**Results:**

Intra-rater ICC(1,1) spanned 0.860–0.973, with SEM 0.14–0.39 mm (MDC95 0.39–1.09 mm). Using two-session averages, inter-rater ICC(2,1) was found to be 0.872, with SEM 0.30 mm (MDC95 0.82 mm). Systematic measurement biases were observed, while more experienced raters showed narrower error ranges. The mean medial elbow joint space width in healthy elbows was 2.8 ± 0.8 mm (range 1.5–4.8 mm).

**Conclusions:**

In this study of static ultrasound images from healthy elbows, intra-rater variability was found to be small, while inter-rater agreement was good among seven orthopedic surgeons with varying experience. These findings reflect measurement reproducibility under standardized static image conditions, independent of image acquisition variability. For practical interpretation, an inter-rater threshold of approximately 0.8–0.9 mm was required to exceed the measurement noise in this dataset, supporting the use of two-session averaging when feasible. Because the present study only evaluated the measurement component, these results likely represent the upper limit of achievable reliability in clinical practice.

## Background

Ultrasound examination is an excellent technique for repeated examinations in athletes because it is minimally invasive, real-time, repeatable, and does not involve radiation exposure [1]. The portability of ultrasound equipment further enables on-field evaluation, thus facilitating primary screening without requiring athletes to visit medical facilities, allowing for immediate assessment and early detection of injuries directly on the field. Recent technological advances have enabled the visualization of finer anatomical details, thereby expanding its application in orthopedic practice [2, 3].

However, a major limitation of ultrasound examination is the variability of results depending on the operator’s skill and experience [4, 5]. Unlike computed tomography (CT) and magnetic resonance imaging (MRI), in which images are captured and then interpreted, ultrasound requires the operator to acquire and interpret the images in real time. It is also difficult to establish standard values for the ultrasound measurement. This operator dependence underscores the need to quantify reproducibility under defined conditions [6].

Currently, ultrasound-based joint space measurement faces two major challenges. First, image acquisition is technically demanding and requires substantial experience. Second, measurement standardization is insufficient, leading to significant variability in assessments between operators. Although some studies have discussed intra-rater variability in joint space measurements, such studies remain limited [7, 8]. However, previous studies on medial elbow joint space have largely emphasized changes under valgus stress or pathological conditions, with limited data on reproducibility or normative static values. This lack of baseline data hampers direct clinical interpretation and comparison across studies. As such, enhancing measurement reliability is essential for consistent interpretation.

Ultrasound measurement of the medial joint space width has been established as an important evaluation method for assessing ulnar collateral ligament (UCL) injuries in the elbow of baseball players [9, 10]. In particular, measuring the changes in joint space width between resting and valgus-stressed conditions has been frequently reported as a method to assess UCL instability [4, 7]. However, reliable data under controlled, static evaluating conditions—reported with explicit error metrics—remain limited. The present study addresses this issue of reproducibility in static-image readings under controlled conditions.

Based on this background, we conducted the present study to quantitatively evaluate operator-dependent variability in static ultrasound image interpretation, focusing on medial elbow joint space measurements of healthy elbows. This study thus aims to establish measurement accuracy parameters, including the minimum detectable change (MDC) in healthy individuals, with the aim of providing standard values for the future clinical application of ultrasound-based evaluations of medial elbow joint space.

## Methods

### Study design and participants

This cross-sectional observational study evaluated the intra- and inter-rater agreement of medial elbow joint space measurements using ultrasound images. Target images were extracted from an existing dataset of ultrasound images of healthy elbows. The study was conducted in accordance with the principles of the Declaration of Helsinki, and received approval from the Ethics Committee of the Institute of Medicine, University of Tsukuba (approval number: 1517-5) and the Ethics Committee of the Center for Computational Sciences, University of Tsukuba (approval number: 23 − 004). Written informed consent was obtained from all participants.

### Ultrasound examination protocol

All ultrasound images were acquired in the standardized limb position reported in prior studies: supine position, 90° shoulder abduction and external rotation, 90° elbow flexion, and a neutral forearm position [7]. All ultrasound examinations were performed by the same examiner in a single standardized limb position, and both the dominant and non-dominant sides were included.

Ultrasound imaging was conducted using a SONIMAGE MX1 system, equipped with a linear probe L3-L11 MHz (KONICA MINOLTA JAPAN Inc., Tokyo, Japan). All images were saved in DICOM format, converted to BMP format, and managed as static images. Image data were managed using Labelbox software (Labelbox Inc., San Francisco, CA, USA), and only static images were used for measurement analysis. The depth was set to 3.0 cm, and the focus was adjusted to the level of the joint space.

We continuously scanned and recorded the medial elbow; from the images that depicted the joint region along a line extending from the apex of the medial epicondyle toward the ligament, we extracted those that satisfied the inclusion and exclusion criteria outlined below. During acquisition, continuous cine clips of the medial elbow were recorded while slowly sweeping the probe to optimize depiction. Ten distinct frames at different time points were extracted as static images from each elbow. All final selections were made by the same examiner based on the predefined inclusion and exclusion criteria described below.

#### Inclusion criteria

Images were included if they met the following conditions: (1) allowed clear visualization of bone edges extending from the medial humeral epicondyle to the ligament attachment; (2) allowed clear visualization of the joint space, specifically the medial humeral trochlea surface and proximal ulna; (3) allowed distinct observation of ligament fiber patterns; and (4) with minimal artifacts that could interfere with measurement.

#### Exclusion criteria

Images were excluded if: (1) the patient had a history of fracture or surgery; (2) there was a prior diagnosis of baseball-related elbow injuries; or (3) the image quality was insufficient to allow reliable identification of anatomical landmarks.

Based on these criteria, 130 high-quality images (10 images from each of 13 elbows) were finally selected for analysis. All ultrasound images were reviewed and selected for inclusion by a single experienced orthopedic surgeon according to the predefined inclusion and exclusion criteria to ensure consistent image quality across all samples.

### Grouping of raters

Seven orthopedic surgeons with varying levels of ultrasound examination experience participated in the study (Table 1). All raters specialized in upper-limb surgery and routinely performed ultrasound-guided procedures and musculoskeletal ultrasound evaluations. No additional training sessions were conducted specifically for this study; each rater performed measurements based on their own clinical experience. For supplementary analysis, raters were classified into three groups based on their ultrasound examination experience: Group A (≥ 5 years of ultrasound experience, 2 raters), Group B (2–5 years of ultrasound experience, 3 raters), and Group C (< 2 years of ultrasound experience, 2 raters). Years of experience included both image interpretation and actual performance of ultrasound examinations and guided interventions. Ultrasound experience was defined as the total number of years engaged in both image acquisition and diagnostic interpretation in clinical musculoskeletal practice.

**Table 1:** Baseline characteristics of the participating raters

Group	Rater	Orthopedic experience (years)	Ultrasound experience (years)
A	A-1	18	10
A	A-2	12	7
B	B-1	15	4
B	B-2	13	3
B	B-3	10	4
C	C-1	8	2
C	C-2	4	0

### Measurement protocol

**Fig. 1:**
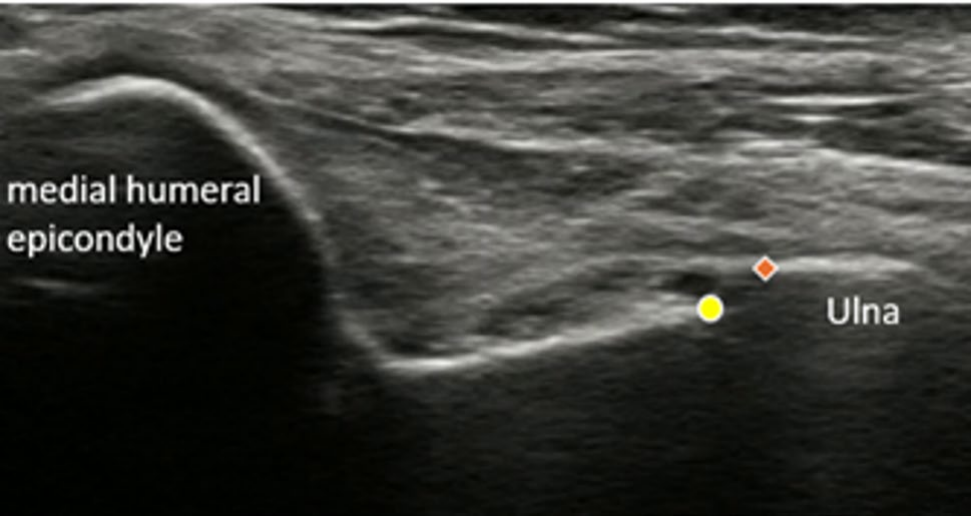
Representative ultrasound image showing medial elbow joint space measurement. The humeral side of the joint space is marked with circles (○), and the ulnar side is marked with diamonds (◇)

The joint space width was measured using the following standardized procedure (as outlined in Fig. 1):

1. Image preparation: All 130 images were randomized once, and this identical sequence was used for both measurement sessions. All identifying patient information and acquisition dates were anonymized.
2. Measurement method: Each rater measured the joint space width at the medial elbow joint space, defined as the narrowest distance between the distal medial edge of the humeral trochlea and the proximal edge of the ulnar trochlear notch. Raters were instructed to identify and measure what they considered the most representative joint space width based on their clinical experience, without any specific anatomical landmarks being specified. This approach was intentionally chosen to reflect real-world clinical practice, in which slight variations in measurement location contribute to inter-rater variability. All measurements were conducted using Labelbox software for coordinate-based pixel calculation, with subsequent conversion from pixels to mm based on the image calibration data. No additional instruction or training session was conducted before measurement; each rater performed the task independently based on their own clinical experience.
3. Measurement environment: All measurements were conducted using participants’ own computer monitors, with image magnification and viewing settings left to individual preference to reflect real-world clinical practice. Therefore, minor differences in monitor size and resolution among raters may have existed; this variability was considered acceptable as part of the real-world measurement conditions and was acknowledged as a study limitation.
4. Repeated measurements: Each rater assessed the entire image set twice, with an interval of ≥ 2 weeks, to evaluate intra-rater reliability.
5. Blinding: Raters were blinded to their previous measurements and to the results of other raters. Furthermore, inter-rater blinding was strictly maintained throughout the study.

### Statistical analysis

Statistical analyses were descriptive; measurement error was quantified by the standard error of measurement (SEM), derived from variance components and minimal detectable change at 95% confidence level (MDC95 = 1.96 × √2 × SEM), with all metrics reported in millimeters.

### Reliability analysis

For intra-rater reliability, we calculated the ICC(1,1) using a one-way random-effects model (images as random-effects). In the supplementary analysis, we compared ICCs between experience groups. Inter-rater agreement was assessed using ICC(2,1), computed from a two-way random-effects model with absolute agreement for single measurements, in which the mean of two measurements per rater was used for each image.

### Accuracy assessment

For each image, we calculated the absolute difference between each rater’s measurement and the study-wide reference value (the mean across raters based on their two-session average). We subsequently computed the mean error (signed difference indicating measurement bias) and the mean absolute error (MAE, indicating measurement accuracy) per rater. Following the procedure of Koo and Li (2016), ICCs were interpreted as poor (< 0.50), moderate (0.50–0.75), good (0.75–0.90), and excellent (> 0.90) [11].

### Normative value calculation

In addition, we calculated the mean, standard deviation, and range of the medial elbow joint space width across all raters and sessions to provide baseline normative values in healthy elbows.

For exploratory purposes, inter-group differences in ICC values were evaluated using both one-way ANOVA and the nonparametric Kruskal–Wallis test, followed by Dunn’s test for multiple comparisons.

All analyses were performed using SPSS version 29 (IBM Corp., Armonk, NY, USA).

## Results

### Intra-rater reliability

**Fig. 2:**
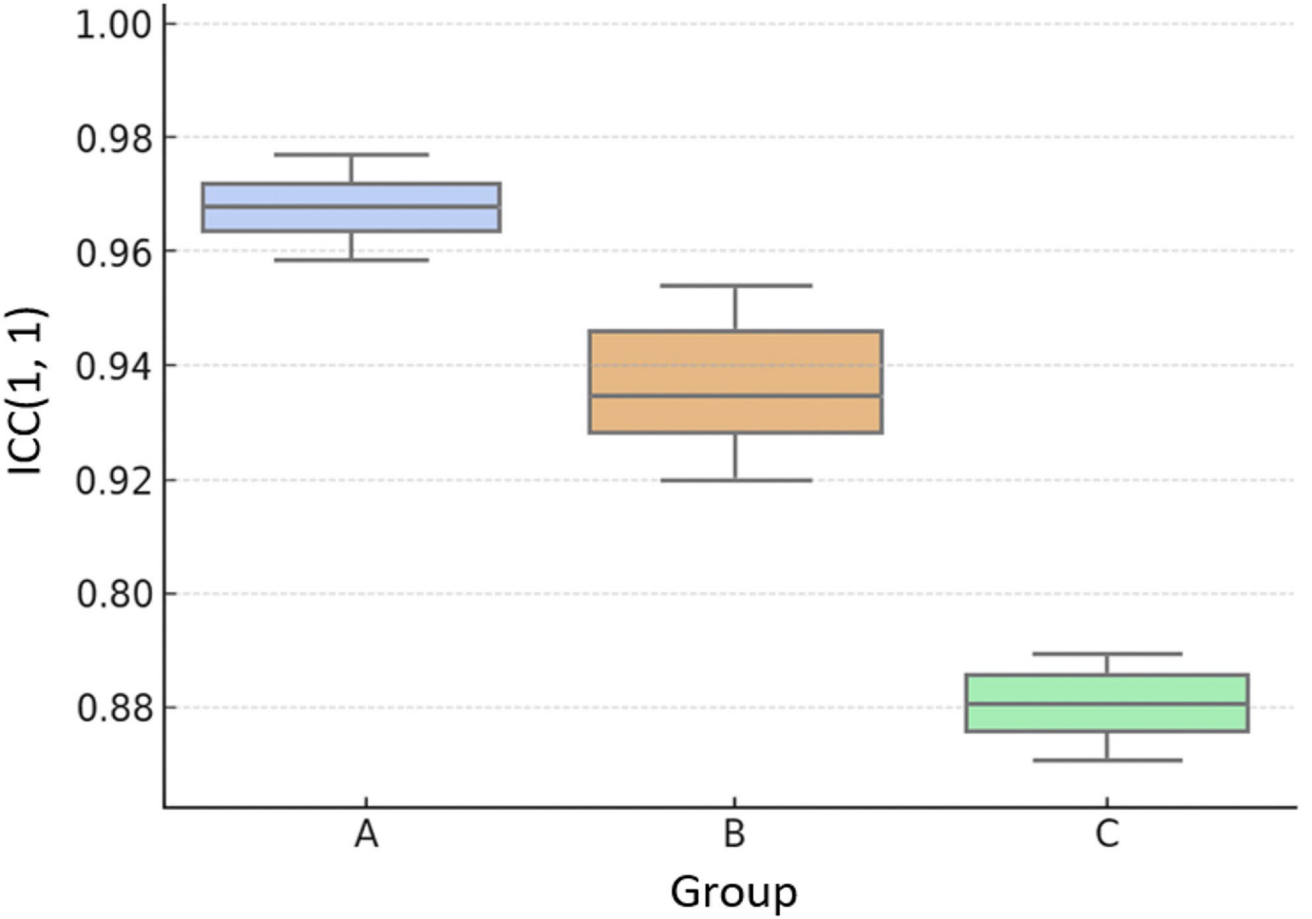
Intra-rater Reliability (ICC(1,1)) by experience group. Box plots showing the distribution of ICC(1,1) values across the three experience groups for descriptive comparison

ICC(1,1) was calculated based on two measurements taken by each rater. All raters demonstrated ICC(1,1) values ranging from 0.860 to 0.973, indicating good to excellent reproducibility (Table 2). Supplementary analysis by experience groups showed mean ICC values of 0.966 (Group A), 0.932 (Group B), and 0.870 (Group C), with descriptive trends suggesting potential differences based on experience level (Fig. 2).

**Table 2:** Intra-rater reliability

Group	Rater	ICC(1,1)	95% CI	Interpretation
A	A-1	0.957	0.940–0.970	Excellent
A	A-2	0.974	0.963–0.981	Excellent
B	B-1	0.932	0.905–0.951	Excellent
B	B-2	0.914	0.881–0.939	Excellent
B	B-3	0.950	0.930–0.964	Excellent
C	C-1	0.860	0.807–0.899	Good
C	C-2	0.879	0.833–0.913	Good

*ICC* Intraclass correlation coefficient, *CI* Confidence interval

ICCs were interpreted as poor (<0.50), moderate (0.50–0.75), good (0.75–0.90), and excellent

Group A (≥5 years of ultrasound experience, 2 raters), Group B (2–5 years of ultrasound experience, 3 raters), and Group C (<2 years of ultrasound experience, 2 raters)

### Inter-rater reliability

Inter-rater agreement was evaluated using the mean of the two measurements per image from each of the seven raters. The resulting ICC(2,1) was 0.872 (95% CI: 0.819–0.905), indicating good agreement among raters.

### Individual rater measurement patterns and errors

**Fig. 3:**
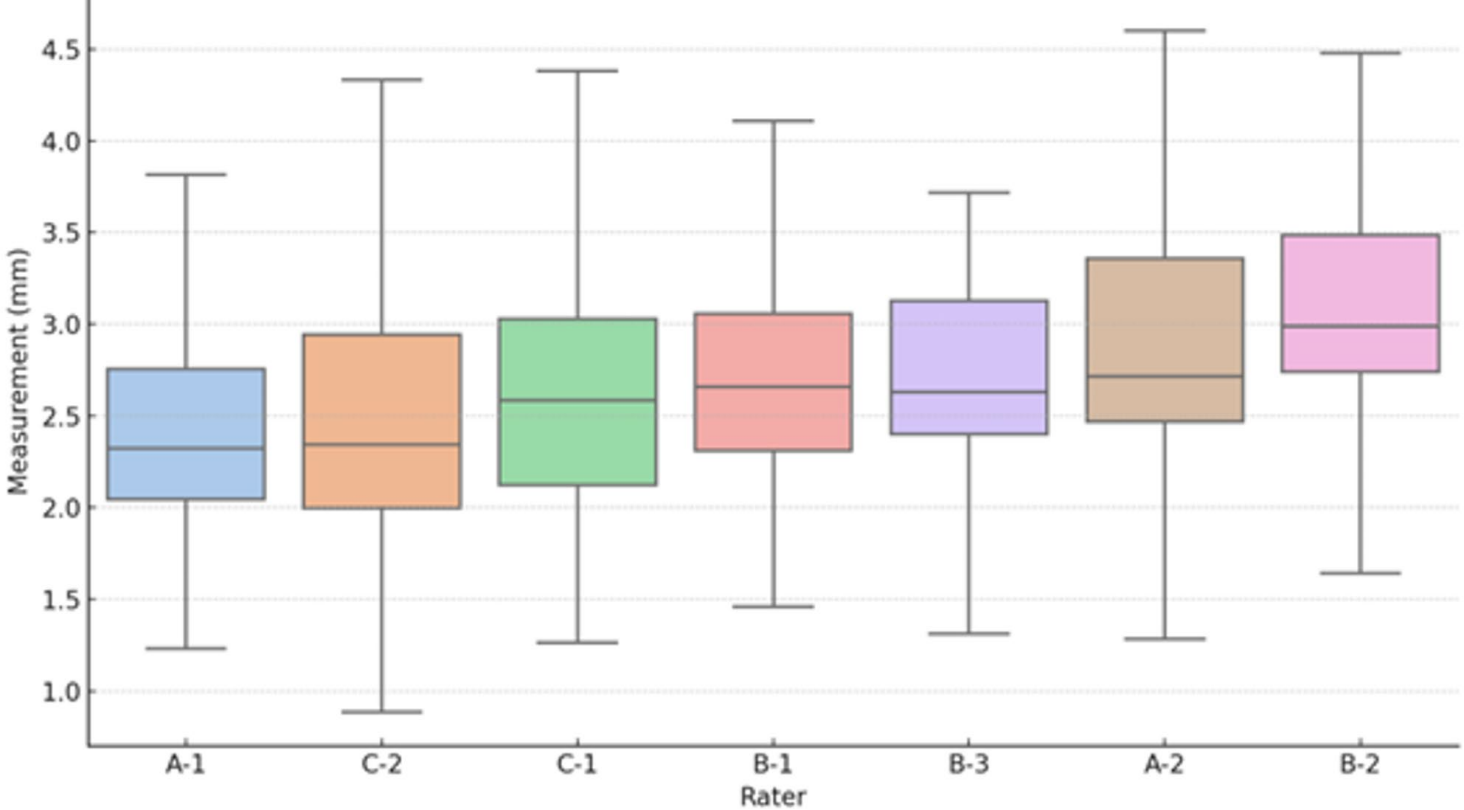
Inter-rater measurement comparison showing individual measurement values by rater. Box plots display the distribution of joint space measurements across all 130 images: boxes indicate the median and interquartile ranges, with whiskers extending to 1.5 times the interquartile range. Outliers are shown as individual points

Using the overall mean measurement value as the reference, individual rater biases were evaluated by calculating the mean errors. Both positive and negative systematic biases were observed (Table 3). The MAE ranged from 0.11 to 0.38 mm. Some raters, such as B-3, showed high reproducibility but large deviations from the reference value, while others, such as A-1, demonstrated smaller absolute errors despite consistent directional bias. These results highlight the importance of assessing not only reproducibility, but also systematic error in individual measurement patterns (Fig. 3).

**Table 3:** Individual rater measurement values and bias

Group	Rater	Mean ± SD (mm)	Bias* (mm)	MAE† (mm)
A	A-1	2.54 ± 0.82	−0.28	0.29
A	A-2	2.94 ± 0.83	+ 0.12	0.17
B	B-1	2.83 ± 0.82	+ 0.02	0.13
B	B-2	3.19 ± 0.86	+ 0.38	0.38
B	B-3	2.85 ± 0.84	+ 0.04	0.11
C	C-1	2.77 ± 0.93	−0.04	0.15
C	C-2	2.58 ± 0.93	−0.24	0.31

*Bias = Individual mean − Overall mean

†*MAE* Mean absolute error from the overall mean

*SD* Standard deviation, *MAE* Mean absolute error

Group A (≥5 years of ultrasound experience, 2 raters), Group B (2–5 years of ultrasound experience, 3 raters), and Group C (<2 years of ultrasound experience, 2 raters)

Measurement error (SEM and MDC95) was also assessed. The intra‑rater SEM ranged from 0.14 to 0.39 mm across raters, corresponding to MDC95 values of 0.39–1.09 mm. For the inter‑rater analysis based on two‑session averages, the SEM was 0.30 mm, corresponding to MDC95 of 0.82 mm. These unit‑based thresholds represent the expected random error for repeated measurements and the minimal change needed to exceed measurement noise (Table 4).

**Table 4:** Measurement error metrics derived from variance components

Outcome	SEM* (mm)	MDC95† (mm)
Within‑rater (across raters)	0.14–0.39	0.39–1.09
Between‑rater (overall)	0.30	0.82

*SEM = Standard error of measurement

†MDC95 = Minimal detectable change at 95% confidence: 1.96 × √2 × SEM

### Descriptive reference values of medial elbow joint space width

Across all raters and sessions, the mean medial elbow joint space width in healthy elbows was 2.8 ± 0.8 mm (range: 1.5–4.8 mm). These descriptive data provide a reference baseline for future comparison with pathological conditions. Supplementary analysis by experience groups showed mean ICC values of 0.966 (Group A), 0.932 (Group B), and 0.870 (Group C).

Exploratory statistical testing indicated a potential difference among the three groups (one-way ANOVA, *p* = 0.0085; Kruskal–Wallis test, *p* = 0.0286), although post-hoc multiple comparisons did not show any significant pairwise differences, likely due to the very small sample size per group.

## Discussion

This study quantified the reliability of medial elbow joint space measurements on static ultrasound images from healthy elbows. Seven orthopedic surgeons with varying ultrasound experience (0–10 years) measured 130 high-quality images twice under controlled conditions, exclusively focusing on the analytical (measurement) component. Intra-rater reliability was excellent across all experience levels (ICC: 0.860–0.973), and inter-rater agreement was good (ICC: 0.872). However, systematic measurement biases were observed among individual raters, with some consistently measuring higher or lower than the overall mean. Descriptive analysis by experience groups showed mean ICC values of 0.966 (Group A), 0.932 (Group B), and 0.870 (Group C), suggesting potential experience-related trends, though the small sample size per group (*n* = 2–3) precludes drawing definitive conclusions. Although this study demonstrated high reliability, the results likely represent an upper boundary for what could be expected in clinical practice. Despite the use of static, high-quality images selected based on strict inclusion criteria, descriptive trends suggested potential differences in reliability based on years of experience. In actual clinical settings, reliability may be further reduced because of several factors, including individual differences in probe manipulation and image acquisition technique, the cognitive burden of simultaneously acquiring and interpreting real-time images, patient-related challenges such as difficulty maintaining position or anatomical variability, and environmental constraints like time pressure or variations in examination settings. Therefore, the ICC values reported here should be interpreted as representing optimal measurement conditions, and lower reliability should be expected in routine clinical practice. These findings emphasize the importance of standardizing ultrasound examination protocols and implementing structured quality management. Furthermore, the systematic biases observed among individual raters may reflect either measurement error or valid alternative interpretations of anatomical landmarks, as no gold standard reference was available for comparison. The relatively narrow confidence interval for inter-rater agreement (0.819–0.905) demonstrates consistent measurement performance across different operators. While previous studies have established pathological thresholds of > 5.6 mm under valgus stress in professional pitchers, compared to normal resting values of 4.0–5.3 mm [9], validation of our measurement approach in pathological conditions would be necessary to establish clinical utility.

The observed patterns are consistent with principles from deliberate practice theory, which emphasizes structured repetition and conscious feedback are key to developing professional skill improvement [12]. Recent simulation studies suggest that beginners may reach a performance plateau after approximately 15 practices cases [13]. For procedures involving fine anatomical detail, such as joint space measurement, accumulated experience allows for technical stability through consistent probe pressure, standardized angulation, rapid and accurate anatomical identification, and internalization of precise measurement point criteria. These capabilities all help reduce cognitive load and contribute to high intra-rater reliability.

The descriptive trends observed among experience group showed numerical differences, with Group C (novice) demonstrating lower mean ICC values than Groups A and B. However, given the small sample size (2–3 raters per group), these observations should be interpreted cautiously and require validation in larger studies before drawing conclusions about experience-related performance patterns.

These reliability parameters provide baseline measurement data for healthy elbows, though validation in pathological conditions would be necessary before clinical application to disease assessment [1, 7, 14, 15].

A key strength of this study lies in its quantitative assessment of operator dependence in elbow ultrasound and its analysis of how measurement variability correlates with years of experience. Although musculoskeletal ultrasound is widely acknowledged as operator-dependent [1, 5], and joint space measurement reliability has been addressed in prior research [4, 7, 8], few studies have quantitatively examined whether measurement errors systematically vary with experience [16]. This study adds essential knowledge to the field by focusing not on diagnostic outcomes but on measurement precision and the potential sources of error in MSUS applications. Additionally, intra-rater reliability was assessed through repeated measurements performed with an interval of at least two weeks, minimizing memory bias. This methodological rigor enhanced the internal validity, facilitating a more accurate assessment of reproducibility.

Several limitations should be noted. First, this single-center study involved orthopedic surgeons from similar training backgrounds, which may limit generalizability across different clinical settings and specialties. Second, this study focused on the reliability of measurement (analysis) rather than on image acquisition. We used pre-acquired static images to isolate the analytical component of operator dependence. However, in real clinical practice, clinicians determine limb position, acquire and select images, and then perform measurements. Each of these steps introduces additional variability; therefore, the reliability observed here likely represents the upper bound compared with live scanning conditions. Third, all measurements were conducted on extracted static images in a laboratory setting, rather than during live patient examinations. This separation from the clinical environment may reduce ecological validity, as real-time ultrasound requires simultaneous image acquisition and interpretation under patient- and environment-related constraints. Fourth, all raters performed measurements on their own computer systems, so monitor size, resolution, and brightness could not be completely standardized, although all were within typical clinical ranges. Fifth, we used a consensus-based mean value as the reference standard instead of an absolute gold standard, limiting our ability to assess absolute measurement accuracy.

Future research should include multicenter validation with raters from various specialties and experience levels, as well as comparisons under real-time scanning conditions. Given that this study demonstrated quantifiable measurement variability even under optimal static image conditions, the development of automated or AI-assisted measurement tools may help minimize observer-dependent variability and further standardize quantitative musculoskeletal ultrasound assessment.

## Conclusions

Overall, this study examined the reliability of medial elbow joint space measurements among orthopedic surgeons using static ultrasound images of healthy elbows. The results of our analysis showed that intra-rater reliability was consistently high (ICC: 0.860–0.973) across all experience levels, inter-rater agreement was good (ICC = 0.872), though systematic measurement biases were observed among individual raters. We established measurement precision parameters including MDC95 values of 0.39–1.09 mm (intra-rater) and 0.82 mm (inter-rater). These findings demonstrate quantifiable operator-dependent variability in ultrasound-based elbow joint space measurement, despite overall good reliability. Moreover, the normative medial elbow joint space width in healthy elbows was 2.8 ± 0.8 mm, providing descriptive reference data for future clinical and research comparisons.

## Data Availability

Anonymized data underlying the results are available from the corresponding author upon reasonable request.

## Abbreviations

CT: computed tomography
ICC: intraclass correlation coefficients
MAE: mean absolute error
MDC95: minimal detectable change at 95% confidence
MRI: magnetic resonance imaging
SEM: standard error of measurement
MDC: minimal detectable change
UCL: ulnar collateral ligament
